# A toolbox for the retrodeformation and muscle reconstruction of fossil specimens in Blender

**DOI:** 10.1098/rsos.220519

**Published:** 2022-08-24

**Authors:** Eva C. Herbst, Luke E. Meade, Stephan Lautenschlager, Niccolo Fioritti, Torsten M. Scheyer

**Affiliations:** ^1^ Palaeontological Institute and Museum, University of Zurich, Zurich, Switzerland; ^2^ School of Geography, Earth and Environmental Sciences, University of Birmingham, Birmingham, UK; ^3^ Department of Cell and Developmental Biology, University College London, London, UK

**Keywords:** blender, reconstruction, retrodeformation, modelling, fossils, muscles

## Abstract

Accurate muscle reconstructions can offer new information on the anatomy of fossil organisms and are also important for biomechanical analysis (multibody dynamics and finite-element analysis (FEA)). For the sake of simplicity, muscles are often modelled as point-to-point strands or frustra (cut-off cones) in biomechanical models. However, there are cases in which it is useful to model the muscle morphology in three dimensions, to better examine the effects of muscle shape and size. This is especially important for fossil analyses, where muscle force is estimated from the reconstructed muscle morphology (rather than based on data collected *in vivo*). The two main aims of this paper are as follows. First, we created a new interactive tool in the free open access software Blender to enable interactive three-dimensional modelling of muscles. This approach can be applied to both palaeontological and human biomechanics research to generate muscle force magnitudes and lines of action for FEA. Second, we provide a guide on how to use existing Blender tools to reconstruct distorted or incomplete specimens. This guide is aimed at palaeontologists but can also be used by anatomists working with damaged specimens or to test functional implication of hypothetical morphologies.

## 1. Introduction

Advances in computer modelling have provided several useful computational tools for anatomists and palaeontologists [1]. Physics-based methods such as finite-element analysis (FEA) [2], computational fluid dynamics (CFD) [3] and multibody dynamics [4] enable researchers to investigate the functions of anatomical structures in both extinct and extant taxa. However, such methods require complete, undistorted, clean meshes of the skeletal structure of interest, as well as muscle force magnitudes and directions. Fossils are often distorted and incomplete, and muscles are typically not preserved in extinct animals. Even in human anatomical studies, creating three-dimensional muscles, for example for patient specific models, can be time consuming and difficult with suboptimal *in vivo* scan data. The aim of this paper is to present new modelling methods for muscle reconstructions and skeletal retrodeformations using Blender.

In palaeontological studies, Blender has been most frequently used for rendering high-quality images as it is a powerful ray-tracing software package. Fairly few publications have taken advantage of the power of Blender for mesh creation, manipulation, positioning and analysing range of motion [5–12]. The use of three-dimensional data to study fossil material is currently an increasingly popular and dynamic field of research including biomechanical, finite element and geometric morphometric analyses exploring taxonomic and ecological questions, all predicated on digital three-dimensional models. After an initial learning curve, Blender delivers versatile and user-friendly tools to work with three-dimensional data, for example to reconstruct muscle morphology.

The reconstruction of musculature for fossilized specimens (in particular of jaw closing muscles) predates modern computational methods. Traditional attempts have used simplified line drawings and force vectors or have been restricted to the identification of muscle attachment sites on the fossilized bones (e.g. Lull [13], Adams [14], Haas [15], Barghusen [16]. Thomason [17] developed the ‘dry skull method’, in which he measured the available cross-sectional area for muscles based on the surrounding bones, then multiplied this by a maximum tension estimate to approximate bite force (which was then corrected based on *in vivo* values). Although three-dimensional muscle reconstructions using physical models have also been created of fossil vertebrates [18], the advent of digital visualization and modelling techniques have facilitated the reconstruction of digital muscle models.

Similar to traditional approaches, digital muscle reconstructions of fossils usually start with estimates of muscle origins and insertions, which can be reconstructed using osteological correlates and extant phylogenetic bracketing [19–21]. Muscle forces are obtained by calculating cross-sectional area, which in turn is typically calculated from muscle volume. However, in contrast to the approach of Thomason [17], the cross section is usually calculated by dividing the three-dimensional volume of the muscle by its length, rather than choosing one area to measure cross-sectional area. To obtain muscle volumes for force calculations, studies have approximated muscle volumes as frustums (partial cones) spanning between the origin and attachment area [22,23]. Another method is to use modified NURBS (non-uniform rational B-splines) circles (essentially designating the shape of several sections along the muscle) and bridging between these, which requires the user to know the positions and shapes of specific cross sections along the muscle [24]. Demuth *et al.* [25] developed an approach based on extruding edge loops to generate the muscle volume. Other studies [26–29] have created detailed three-dimensional muscle volumes by drawing on muscles in computed tomography (CT) data stacks (in slice view before three-dimensional model creation), which prevents intersections of muscles and bones. However, it is not intuitive or time efficient to draw muscles in two-dimensional views. Furthermore, this approach requires access to specialist (and often commercial) software to process tomographic datasets, which restricts more widespread accessibility.

Comparisons of two-dimensional muscle attachment reconstructions (measured from lateral photographs) and three-dimensional muscle attachment reconstructions in carnivorans demonstrated that the three-dimensional methods correlated equally or more closely with muscle mass and physiological cross-sectional areas (PCSA) from dissections than did the two-dimensional measurements. Furthermore, estimates of maximum cross-sectional area from photographs (‘Thomason dry skull method’) were more closely related to PCSA obtained from dissections than using muscle attachment areas [30]. Note that in the Dickinson study, origin and insertions were compared separately, whereas the frustum approach (e.g. Sellers *et al.* [22], Wilken *et al.* [23], Cost *et al.* [19]) takes into account both origin and attachment areas, as well as muscle length. Bates *et al.* [31] compared the Thomason [17] dry skull method and an approach based on attachment areas and found that for both methods, muscle properties, bite force, bone stress and stress patterns were less accurate compared to data and models obtained from cadaver data.

Modelling muscles in three dimensions has the potential to yield more accurate muscle forces, since muscle forces are often estimated from cross-sectional area (which is often obtained by dividing volume by length) in reconstructions of fossils.

To reconstruct muscles, an undistorted, complete three-dimensional model of the organism of study is needed. Three-dimensional models of fossil specimens derived from photogrammetry, surface scanning, CT-scanning and other techniques have become common in palaeontological studies. However, before any form of soft-tissue reconstruction can be attempted, the studied specimens need to be restored to an ‘*in vivo*’ condition. Preservation of fossil material is universally affected by taphonomy. For example, fossil specimens are often distorted, disarticulated and/or possess cracks. Furthermore, sometimes only the element from one side is preserved. It is often important to correct morphological distortions, termed retrodeformation, before analyses are possible [26,27,32–34]. As a further complication, some analyses (such as FEA or osteological range of motion studies) as well as three-dimensional printing require ‘watertight’ meshes (i.e. ‘manifold’ meshes, without any holes or other geometric problems such as faces with 0 area, etc.) [33,35–37]. Mesh cleaning and remeshing can also be performed in Blender.

Here, we developed a new interactive method in Blender to model three-dimensional muscle volumes based on a user’s knowledge of the muscle origin and insertion sites. We also provide a practical guide for retrodeforming fossil specimens in Blender. Blender is a free, open source software with integrated Python scripting. Our Python code was wrapped as a Blender add-on to enable easy, straightforward use of the tool.

## 2. Methods

We developed our methods in Blender (v. 2.91.0–2.93.0), a free software licensed under the GNU general public licence. The add-on currently works for v. 2.91.0–2.93.0.

Blender is a free and open-source software package, usable on Windows, Mac and Linux. It is a versatile and accessible tool for the creation, manipulation and presentation of three-dimensional data. At the time of writing, Blender is on v. 3.10, and continues to be a software package with frequent and significant updates and changes, including significant changes to the user interface introduced with v. 2.80. Many features introduced in these updates have current and potential palaeontological uses.

### 2.1. Blender add-on for reconstructing muscles

#### 2.1.1. Add-on information

**Figure 1.**
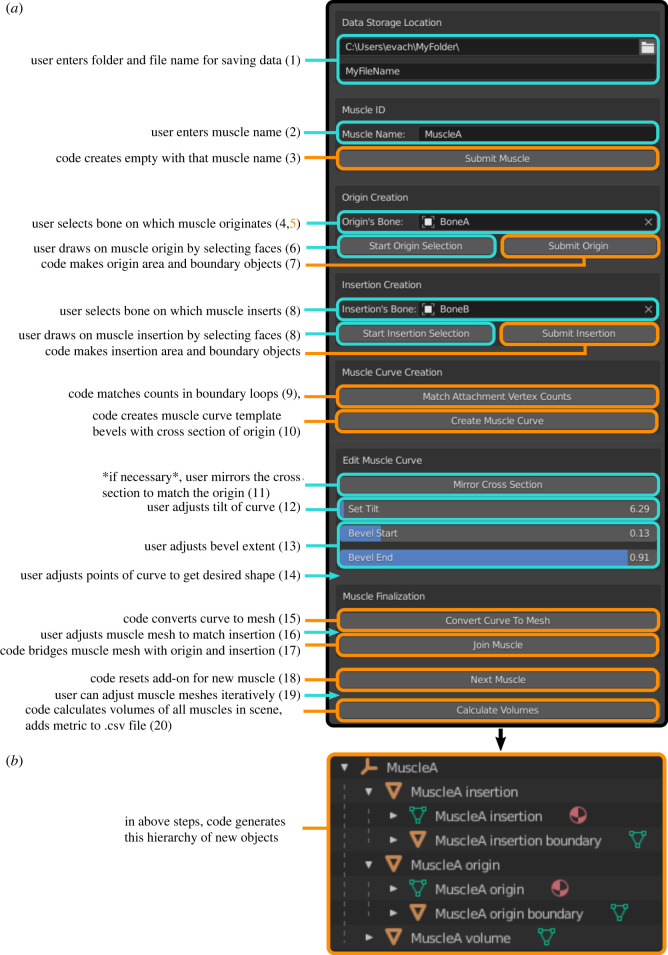
(*a*) Steps of the Blender add-on and (*b*) object outputs in hierarchy view. Cyan colour indicates user input, orange colour indicates operations automatically performed by the programme. More detailed steps are included in the text, and are references by the numbers.

The muscle reconstruction method was developed using Python script in Blender and wrapped as an add-on to enable user inputs (e.g. muscle names, selection of attachments, adjustment of muscle curvature). The ‘Myogenerator’ add-on starts with the user ‘painting on’ origin and insertion attachment areas. A curve is then created automatically by the code, spanning between centroids of these areas, and the user can adjust the curve. The curve is then automatically bevelled with the shape of the origin boundary (projected into two dimensions) and meshed to facilitate further user adjustments. An automatic reorientation step (aligning the largest dimensions of the origin boundary to the *XY* plane) before the bevelling step ensures that the bevel shape is not affected by the mesh orientation in space. The curve mesh is then automatically joined to the origin and insertion areas, producing a three-dimensional muscle model. More detailed steps are given in figure 1*a* and the README file of the Github repository (see Data accessibility). The ‘Myogenerator’ add-on also automatically organizes the muscles in the scene by names (grouping muscle attachment areas, attachment boundaries and volumes as ‘children’ under each muscle in the object hierarchy; figure 1*b*). The add-on also automatically generates a .csv file with all of the muscle metrics (name, origin area, insertion area, origin centroid, insertion centroid, linear length, muscle length, muscle volume). Linear length is calculated as the Euclidean distance between origin and insertion centroids, muscle length is calculated as the length of the curve of the Blender muscle (the sum of edge lengths constituting the curve). Headers are included, and multiple muscles are written to the same file as rows. To install the add-on, download the Add-on Folder from our Github repository (see Data accessibility), make sure it is zipped and then follow the instructions here (https://docs.blender.org/manual/en/latest/editors/preferences/addons.html).

We tested our muscle reconstruction tool by modelling the muscles of the extinct herbivorous dinosaur *Erlikosaurus andrewsi*. The muscles of *Erlikosaurus* were previously reconstructed using simplified cylinders that were then ‘fleshed out’ using a two-dimensional drawing and interpolation approach in Avizo [28]. Our goal was to use this previous study as a reference for the morphology of the muscles, to compare our tool to other methods. For the case study, the eight jaw adductor muscles of *Erlikosaurus* were reconstructed. This process was performed by the same person (S.L.) to ensure maximal consistency. S.L. has extensive experience with both software programmes so that the impact of familiarization with the programme on reconstruction duration could be discounted. Muscle definitions and attachment sites follow Lautenschlager [28]. In a first step, each muscle was modelled using the new method to create a base model before further refinement. In a second step, the muscle models were further adjusted using mesh editing and sculpting. Muscle portions which were too thin or too thick were manually adjusted, such as for example the pterygoideus ventralis muscle (mPTv) which has a ventrally bulging morphology in most archosaurs. The curve adjustments in the first step allow some intersections to be avoided (relative to approaches such as frustum approaches, which connect the origin and insertion attachments directly without accounting for muscle curvatures). The muscle models were also manually inspected for any remaining regions of intersection (intersections between muscles and bone and between individual muscles). These muscle portions were adjusted using the Sculpt tool in Blender which allows users to move individual regions, to smooth surfaces and to add material. Furthermore, Boolean intersections were used to automatically check and remove regions of muscle–muscle and muscle–bone contact.

We also used the origin and insertion areas to compare muscle lengths and volumes obtained using the frustum approach [22] with our new method. Our plugin exports the following metrics: three-dimensional Blender volumes, muscle attachment areas and centroids, linear muscle length (origin centroid to insertion centroid), curved muscle length and three-dimensional volume. Frustum volumes were calculated according to Sellers *et al.* [22]:

2.1 $$V_{M}=\frac{l_{M}}{3}\left(A_{\mathrm{or}}+A_{\mathrm{ins}}+\sqrt{A_{\mathrm{or}}\cdot A_{\mathrm{ins}}}\right).$$

PCSA was calculated by dividing muscle volume by the fibre length, assuming that fibre length is equal to muscle length, and assuming a parallel-fibred muscle for simplicity. Holding pennation angles and the relationship between fibre length and muscle length constant enables us to investigate the effects of three-dimensional muscle volumes and curved versus straight muscle length calculations; different pennation angles and muscle fibre lengths would change the absolute PCSAs and forces, but not the calculated relative difference in PCSAs and muscle forces between the two methods tested. Muscle force was calculated by multiplying PCSA by an isometric muscle stress value of 0.3 N mm^−2^ following Thomason [17] and Wroe *et al.* [38].

### 2.2. Tools and guidelines for specimen retrodeformation

Fossil morphologies are often distorted and incomplete, and must be restored before muscles can be reconstructed. Here, we present a number of Blender functions with potential uses in palaeontological retrodeformation, accompanied by examples of their use, to highlight these techniques and explain their use and function. The terminology of this paper does not make a strict distinction between ‘retrodeformation’ and ‘restoration’. An overview of the key digital techniques and methods was given by Lautenschlager *et al.* [32] comprising: fixing cracks and breaks; mirroring, superimposing, repositioning and duplicating elements; and plastic retrodeformation. The tools presented here demonstrate that all of these retrodeformational techniques can be performed in Blender. We encourage readers to take advantage of the accessibility and power of Blender for their own use.

We try to offer an introductory explanation of various techniques, aimed at a level with basic knowledge of Blenders layout, function, controls and terminology. We cover techniques using three different ‘modes’ within Blender. These are: (i) Object Mode—Blender’s default mode, used for object-level edits like translation, rotation, and scaling; (ii) Edit Mode—used to change an object’s shape and structure by editing the vertices/edges/faces of meshes and (iii) Sculpt Mode—Blender’s three-dimensional sculpting tool for changing the shape and structure of an object by changing its mesh with a variety of ‘brushes’. Tools in these modes and the use of Modifiers (automatic operations added to objects to affect their geometry) allow the user to create and manipulate material for retrodeformation. There is no single recommended workflow—every specimen is different, requiring different techniques and considerations.

#### 2.2.1. Guidelines to maintain accuracy and precision

Working in orthographic view keeps objects the same size and proportions regardless of the distance between the viewport camera and the object, allowing for more objective assessment of shape and the use of reference images. Reference images can be added into the main window as objects. Images of undeformed specimens, closely related species or useful schematics, should be used wherever possible to guide creating material from scratch or editing and preserve objectivity and accuracy. The image can be resized, rotated, and translated as needed within the window view space.

Positioning objects in alignment with the main axes allows for effective use of preset orthographic view points along each axis and for constraining mesh edits and sculpting along certain axes, which can increase control and precision. Correct model alignment also allows for easy use of mirroring (Object > Mirror; and the Mirror modifier).

There should be justification for every edit on the geometry of a specimen. The most objective source for material to replace missing areas is from another symmetrical side/area of the specimen, followed by other specimens of the same species, then closely related specimens. Symmetry within biological structures or other indicators (such as orbit shape being roughly circular; Arbour & Currie [39], Cuff & Rayfield [27]) can present objective evidence of deformation.

If surface details are important, multiple remeshing/smoothing may deteriorate them; use these tools sparingly. We recommend to make use of frequent saving and backups so versions prior to edits are not irrevocably lost. Similarly, Blender’s modifiers are beneficial as they are non-destructive—they can be added and saved while being able to be turned on/off without permanent changes to their object until applied.

#### 2.2.2. Creating new three-dimensional material

When material is completely missing or severely damaged and cannot be sourced from elsewhere in the specimen (i.e. mirrored) or material from another individual or species cannot be manipulated to form a replacement, it may be necessary to create new three-dimensional material for restoration using the following techniques. Alternatively, a structure can be created purely by editing a default object (typically a sphere) using Sculpt Mode with dynamic topology (described below, in ‘Editing three-dimensional material’ section).

*Box modelling*. Box modelling is a general method to create new three-dimensional material by repeated addition and modification of simple shapes. Garwood & Dunlop [6] presented an effective guide for box modelling in a palaeontological context and the application of box modelling to palaeontological functional analysis was subsequently explored in detail by Rahman & Lautenschlager [11] in which box-modelled structures performed very similarly to models derived from tomographic or surface-based techniques.

Workflows can include extrusion of individual vertices/edges/faces in Edit Mode as well as moving edges or vertices, gradually building up a shape. It is also possible to create default shapes like cubes, spheres, cylinders, etc. and modify and join them (e.g. using the *Boolean* modifier, described below) to approximate a morphology until a mesh with adequate detail is reached. The *Subdivision Surface* modifier and remeshing are important tools that can increase the face count of a basic structure to allow additional modifications and create detail (see also Sculpt tool Dyntopo setting).

Ultimately, a new morphology can be combined with other elements of the specimen using the *Boolean* modifier and remeshing (described below).

**Skin* modifier*. The *Skin* modifier extrudes a mesh around vertices and edges, ‘fleshing out’ simple strings of vertices. This can be useful, when combined with a *Subdivision Surface* modifier, to rapidly create a basic shape for further editing. Vertices can be quickly added in Edit Mode. The scale of vertices determines the skin thickness and can also be changed in Edit Mode.

An alternative is creating a structure using curves. These can be fleshed out by changing the depth setting in the Bevel tab under Object Data Properties. This can then be converted to a mesh (Object > Convert > Mesh).

#### 2.2.3. Editing three-dimensional material

**Figure 2.**
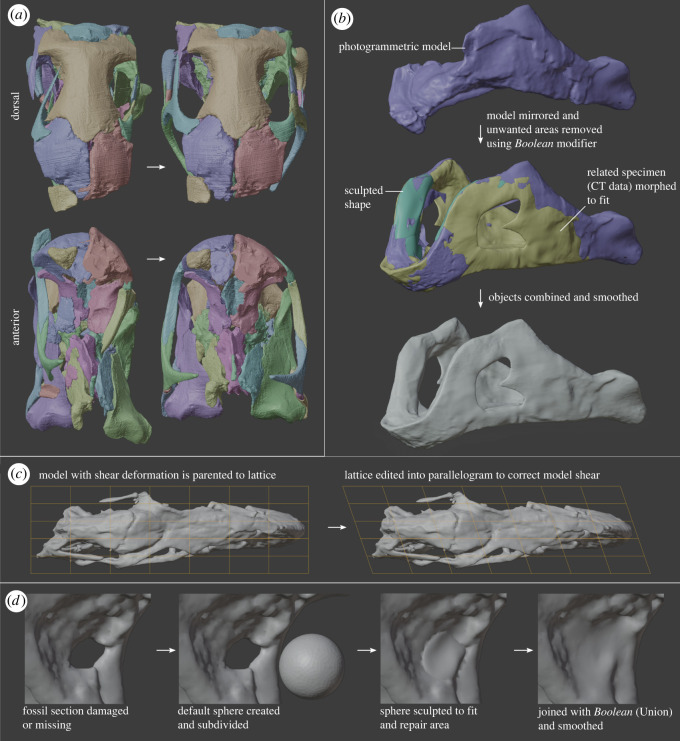
Examples of retrodeformation processes in Blender. (*a*) Elements were correctly repositioned as separate objects; (*b*) combination and smoothing of photogrammetric, CT, and sculpted models to produce a single mesh; (*c*) lattice modifier used to correct model shear and (*d*) sculpting a sphere to reconstruct a missing part of the fossil, followed by a Boolean union modifier to join the meshes.

*Reposition, rotate, scale and combine material in Object Mode*. The repositioning and scaling of elements (sometimes mirrored, see Mirroring below) are common steps in retrodeformation. If different parts of a specimen exist as separate three-dimensional objects (i.e. segmented separately from CT-data or individually modelled with photogrammetry), these can be repositioned in Object Mode (figure 2*a*). Elements can also be isolated into separate objects by duplicating the specimen, then isolating desired elements in Edit Mode, or ‘cutting’ them out with *Boolean* modifier. They can then be moved, rotated, scaled using the main window buttons or with shortcuts (see electronic supplementary material for instructions). Separate elements can be re-joined into one object (Object > Join), then remeshed to combine their meshes, or using the *Boolean* modifier (*Union* setting) then remeshing to fix poor mesh geometry.

*Snap to surface*. These options ‘snap’ object and mesh transformations in relation to other objects’ geometry. For example, setting the ‘Snap to’ option to ‘Vertex’ and the ‘Snap with’ to ‘Closest’ will snap the closest point of the object being moved to the surface vertices of its closest neighbouring object, essentially guiding translation so they don’t intersect, aiding in repositioning elements.

*Edit Mode*. Edit Mode is the default modelling mode in Blender used to edit vertices, edges and faces that comprise three-dimensional meshes. Directly editing the mesh of an object derived from methods like CT-scanning or photogrammetry is difficult due to their complexity (alternate techniques are suggested below, e.g. *Lattice* modifier, Sculpt Mode, remeshing). However, it may be possible and it is relevant when creating and editing material through box modelling or editing other material of a relatively simple geometry (or given a simple geometry, i.e. through remeshing). Direct mesh editing can also be used to fill small cracks, join gaps in a mesh, move and smooth vertices, or otherwise effectively improve small regions of morphology. Vertex smoothing can also be useful to smooth specific regions of the mesh that are irregular due to damage or retrodeformation operations. The proportional editing setting allows transformation of vertices/edges/faces to also affect nearby elements, the accompanying changes being proportional to their distance from the transformed elements (controlled by Falloff type setting). This allows smoother transformations of a dense mesh.

Edit Mode has a number of tools to ‘clean’ meshes, i.e. to help eliminate artefacts and errors. These include tools to automatically correct normals, fill holes, and beautify faces (a Blender tool to reduce the number of long thin triangles by setting a maximum angle threshold). It is important to keep the mesh geometry without errors such as non-manifold edges, intersections and holes. Clean meshes are technically essential for the models to be used in techniques like FEA or CFD.

*Sculpt Mode*. Sculpt Mode features a variety of ‘brushes’ used to alter the topology of mesh areas (figure 2*d*). Sculpting is a more intuitive and effective way of altering the geometry of a complex mesh than Edit Mode. Care must be taken to maintain clean mesh geometry and an objective approach in retrodeformation.

Each sculpting brush has a different effect on its region of influence. The size and intensity of the brush effect can be changed in the tool settings bar. Brush effects are reversed by Ctrl clicking. The *Smooth* brush is available with any brush by Shift clicking. Sculpting can be locked to affect only certain axes, and can be mirrored on specified axes.

*Draw, draw sharp, clay, clay strips* and *Layer*—move vertices inwards/outwards giving the impression of adding and removing material. Each behaves subtly differently.

*Inflate* and *Blob*—inflate/deflate and increase/decrease bulbosity of structures, respectively.

*Smooth*—vital brush to maintain a clean mesh and even out the effect of other brushes (figure 2*b*)

*Grab*—grab an area of a mesh and move it around.

*Elastic deformation*—similar to *Grab*, but smoother and less deleterious to mesh quality. Especially useful to manipulate very thin areas which are too fine for adjustment with *Lattice* deformation modifier.

*Pose*—for posing character models in animation but an effective tool for moving linear structures around a pivot more accurately than the *Grab* or *Elastic deform* brushes while maintaining the shape and thickness of the area. See DeVries *et al.* [5] for a Blender retrodeformation method focusing on posing armatures to correct long bones.

*Dynamic topology (Dyntopo)* is a checkbox that allows brushes to add and remove elements of the mesh, rather than just changing their shape. This allows for smoother, more detailed edits, especially for building up detail on low-resolution meshes. Ensure changes to scale have been applied and Dyntopo has a suitable resolution for the best results. Some sculpt edits may result in a messy mesh geometry (especially aggressive brushes like *Grab*). If these cannot be cleaned up with the Smooth brush or through the use of Dynotpo, the remesh tool (as described below) is very effective at fixing the geometry of the mesh and can be accessed directly at the top of the sculpt window.

*Boolean modifier*. A powerful modifier that performs mesh edits that may be complex to do manually, such as joining objects’ meshes together, or using one object to cut shapes out of another (figure 2*d*). The modifier is applied to an object and an operation is chosen to affect the mesh of the object based on how it is interacting with another specified object. *Intersect*—keep areas of the modified object that intersect with the target object. *Union*—join the mesh geometry of the modified object and the target object, removing any internal intersections between the two (unlike the general ‘join’ command). *Difference*—remove areas from the modified object that intersect with the target object. The *Boolean* modifier is an essential tool for joining material together (*Union*) and for isolating parts of a mesh (*Intersect* or *Difference*). It is very effective for modelling, creating complex shapes using the interaction of a few simple geometric objects and a combination of *Boolean* modifier operations. Like other modifiers, *Boolean* is non-destructive and will not affect the mesh of the object until it is applied. If the *Boolean* modifier gives bad results (e.g. not removing internal geometry for *Union* modifier), make sure the meshes are manifold.

*Remesh*. Remeshing will automatically rebuild the mesh geometry of an object. Remeshing can eliminate minor cracks and breaks in a specimen (i.e. Lautenschlager *et al.* [32]). It is also used to generate a more uniform mesh topology following joining of different elements (figure 2*b*) or the *Boolean* modifier (*Union*). The *Remesh* tool is best accessed at the top of the Sculpt Mode window or in the Object Data Properties tab of a selected object. Voxel Remesh generates a new uniform mesh topology for the object based on a user defined voxel size. Lower voxel size values result in a denser mesh, and minimize loss of fine detail. For cases where a smaller mesh size (i.e. larger voxel size)is necessary, we compared using voxel remesh with the end goal size versus using voxel remesh at a finer resolution and then downsampling via the decimate modifier. The former resulted in more evenly spaced triangles but more loss of small features such as foramina, whereas the latter resulted in better preservation of fine details but triangles of irregular sizes.

The eyedropper tool next to the voxel size can also be used to select a reference mesh for the desired voxel size. Note that the result will have quadratic faces (which can be triangulated in Edit mode, see electronic supplementary material). The Adaptivity setting will reduce the final face count by simplifying geometry where detail is not needed based on the value set. The Preserve Volume checkbox will endeavour to preserve the original volume of the mesh.

Alternatively, there is also *QuadriFlow Remesh* which uses a different algorithm (Quadriflow) to similarly create a quad based mesh. A *Remesh* modifier also exists but is an artefact of earlier Blender versions and in our experience it is less effective than Voxel or QuadriFlow Remesh. The results of remeshing can be subtly affected by an object’s orientation with regard to global axes, and the poles of the objects mesh. In our experience, selecting ‘fix poles’ can solve the issue of ‘step-like’ artefacts in the remeshed object.

Note that modifiers will need to be applied before sophisticated mesh manipulations such as remeshing.

*Mirroring* (*and duplication*). We can take advantage of symmetry in biological structures to replace missing elements. Mirror a specific object by selecting and duplicating it (Object > Duplicate). The duplicate can be mirrored with Object > Mirror, followed by the axis the user wants to mirror across (‘*X*’, ‘*Y*’ or ‘*Z*’).

The modifier *Mirror* is also capable of creating a duplicate object, mirrored in one or more axes. The axes the mirroring is active can be selected with check boxes. The modifier will mirror across the object’s origin, the position of which may need to be changed, or a ‘Mirror Object’ can be selected to act as the centre instead.

A common use is to split a model in half and use the *Mirror* modifier to create a structure with perfect symmetry where edits on one side are actively duplicated on the other (as long as the modifier is currently active, and not yet applied). The Clipping checkbox will prevent vertices moving through the mirror plane when transforming them in Edit Mode. The Merge checkbox option will merge vertices with their mirror counterpart when they occupy the same position (or distance defined by Merge Distance). Applying the modifier will make the mesh changes permanent (so that the new mesh consists of both sides, and edits to one do not change the other). Mirroring settings exist independently in Sculpt Mode for use with sculpting brushes as described above.

*Lattice modifier*. A number of deformation modifiers are effective for correcting plastic deformation like compression or shear (figure 2*c*). The *Lattice* deformation modifier is capable of dramatically improving the morphology of a fossil very quickly and intuitively. It is ideal to adjust the shape of higher density meshes where editing individual elements or using proportional editing is impractical.

A lattice is a cage-like grid of vertices that can be deformed as a guide to apply deformation to another object. Do this by creating (Add > Lattice), scaling and positioning a lattice over all/part of the object intended for deformation in Object Mode. The resolution (number of vertex subdivisions on each axis; *U*, *V*, *W* = *X*, *Y*, *Z*) of the lattice should be edited in its Properties to best suit the object and precision of the deformation intended. Add a *Lattice* modifier to the object, and select the lattice within the modifier inputs. Alternatively, to automatically apply the *Lattice* modifier, shift select the object and then the lattice and parent them with *Lattice* deform (Object > Parent > Object). Adjusting the position of the lattice’s vertices or its scale in Edit Mode will result in a similar deformation to the mesh of the linked object. The algorithm of interpolation used for deformation can be changed in the properties tab (e.g. linear will very closely link changes to the mesh and lattice). Proportional editing can be used for a smoother transition between the transformed vertices and the rest of the lattice. The same lattice can be used to deform multiple object at the same time, by giving each the modifier. As with other modifiers, *Lattice* needs to be applied in order to permanently affect the mesh geometry of an object and allow this to be exported.

*Additional deform modifiers*. *Mesh deform*: Similar to Lattice but any mesh can be set as the guide for shape changes on the target—useful if a shape other than a lattice (e.g. a sphere or cylinder) would be more intuitive for controlling deformation.

*Surface deform*: Similar again to *Lattice* and *Mesh deform* but intended for instances where a two-dimensional surface like a planar grid or another object with no depth is used to control deformation of a target.

*Laplacian deform*: A modifier requiring the user to choose a number of ‘anchor’ vertices (e.g. by creating ‘Hooks’ on each vertex). When one or more of these vertices are repositioned, the modifier keeps the rest of the anchor vertices in fixed positions and rearranges the rest of the object to preserve its original geometric details, based on Laplaician Surface Editing [40], which uses differential coordinates to encode geometric information [41].

*Shrinkwrap*: Shrinks down an object to wrap around onto the surface of another. A number of uses can be found for this modifier, e.g. forming a convex hull over a very fragmentary part of a specimen to restore rough overall shape. For best results, the basic shape being shrunk onto the target should be roughly the correct shape and subdivided to have sufficient faces in its mesh to ‘wrap’ correctly. Significant shape change will result in messy mesh geometry that will require remeshing and/or smoothing. The target of the shrinkwrap must be a single object; multiple target objects may be joined with Object > Join, or the *Boolean* modifier (Union).

## 3. Results

### 3.1. Muscle reconstruction tool

The Blender add-on ‘Myogenerator’ is openly available on Github (see Data accessibility).

**Figure 3.**
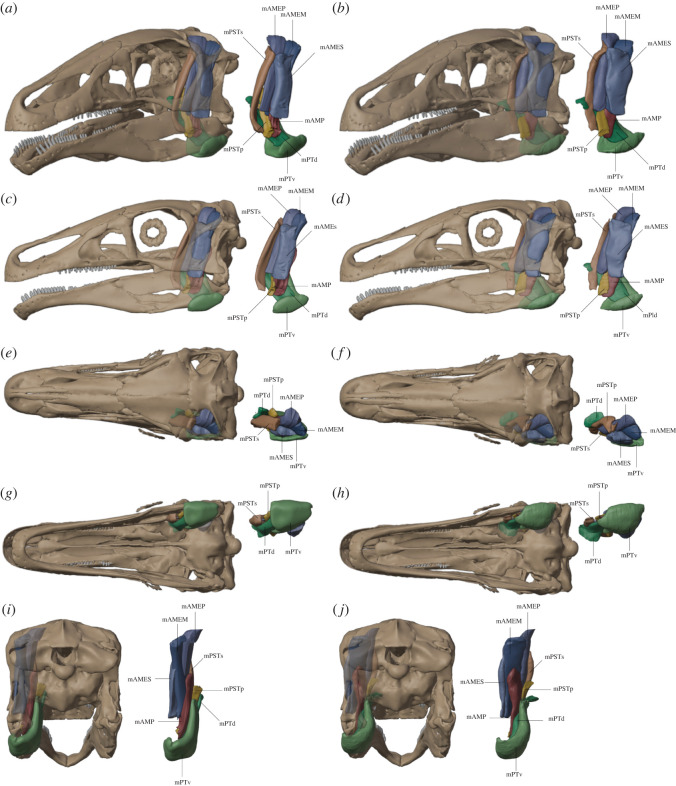
(*a*,*c*,*e*,*g*,*i*) *Erlikosaurus* jaw muscles reconstructed using the new ‘Myogenerator’ Blender tool and (*b*,*d*,*f*,*h*,*j*) muscles reconstructed in Avizo using the slice-by-slice CT modelling approach from Lautenschlager [28]. Muscles in (*a*) were created based on the muscle attachment areas in [28]. (*a*,*b*) Anterolateral view; (*c*,*d*) lateral view; (*e*,*f*) dorsal view; (*g*,*h*) ventral view and (*i*,*j*) posterior view.

A comparison of the two muscle reconstructions (the original Avizo CT-slice based versus the Blender tool) shows largely similar results (figure 3). Both methods were able to achieve similar three-dimensional muscle volumes and shapes. Small differences are likely present in the contact between muscle bodies which can be defined on a slice-level for the CT-based approach compared to the Boolean exclusion approach in Blender.

Time-wise, the CT-based model required an effort of *ca.* 10–15 h of work. In comparison, the Blender-based model took only 2–3 h including subsequent refinement.

Tables 1 and 2 show a quantitative comparison of lengths, volumes, PCSAs, and muscle forces generated from the frustum and Blender approaches for three muscles with a range of morphologies: adductor mandibue externus superficialis (mAMES), adductor mandibulae externus profundus (mAMEP) and pterygoideus ventralis (mPTv). The mAMEP and mAMES could be generated easily from the origin and insertion areas; therefore, the attachments generated during the muscle modelling process were used directly for the frustum calculation. However, the mPTV, due to its wrapping along the jaw, could not simply be bridged to the insertion, requiring more sculpting adjustments to recreate the morphology. Therefore, to ensure that these changes in muscle morphology did not affect the attachment areas, we recalculated these areas and their centroids based on the final muscle morphology to conduct an updated frustum approach. In the Blender three-dimensional muscles, to prevent intersections, the bone–muscle overlap region was subtracted from all muscles. Additionally, because of some overlap in the mAMEP and mAMES, this slight overlap was removed by subtracting the intersecting volume from the mAMES.

**Table 1.** Quantitative comparison of volumes and muscle lengths between frustum and Blender approach for three muscles with a range of morphologies: mAMES, mAMEP and mPTv. All metrics were rounded to the nearest tenth. Per cent differences were measured as (three-dimensional metric - frustum metric)/(three-dimensional metric). Lengths are given in mm, areas in mm^2^, volumes in mm^3^.

muscle name	origin area	insertion area	linear length	muscle length	% diff. muscle length	frustum volume	three-dimensional muscle volume	% diff. muscle volume
mAMEP	187.6	14.5	71.8	76.9	6.5	6086.3	7269.5	16.3
mAMES	113.9	131.0	54.4	60.0	9.4	6651.6	6450.9	−3.1
mPTv (*updated attachments based on three-dimensional volume*)	89.1	569.8	46.5	68.4	32.0	13707.1	11551.1	−18.7
mPTv (*original attachments lateral insertion only*)	76.9	218.3	45.6	68.4	33.4	6451.7	11551.1	44.1

**Table 2.** Quantitative comparison of physiological cross-sectional areas (PCSAs) and muscle forces between frustum and Blender approach for three muscles with a range of morphologies: mAMES, mAMEP, and mPTv. All metrics were rounded to the nearest tenth. Per cent differences were measured as (three-dimensional metric - frustum metric)/(three-dimensional metric). PSCA was calculated by dividing muscle volume by the length, assuming that muscle length equals fibre length and assuming a parallel-fibred muscle for simplicity. Muscle force was calculated by multiplying PCSA by an isometric muscle stress value of 0.3 N mm^−2^, following Thomason [17] and Wroe *et al.* [38]. PSCAs are given in mm^2^, force in Newtons.

muscle name	frustum PCSA	three-dimensional muscle PCSA	frustum force	three-dimensional muscle force	% force difference
mAMEP	84.7	94.6	25.4	28.4	10.4
mAMES	122.4	107.5	36.7	32.3	−13.8
mPTv (*updated attachments based on three-dimensional volume*)	294.7	168.9	88.4	50.7	−74.5
mPTv (*original attachments lateral insertion only*)	141.6	168.9	42.5	50.7	16.2

## 4. Discussion

### 4.1. Retrodeformation

Here, we provide a user guide to applying a range of Blender tools to restoring fossil morphologies in three dimensions. These tools can be extremely useful when adjusting meshes of extinct organisms; however, decisions on the exact method and scope of the process lie on a case-by-case basis. Understanding of different modes of damage (removal of material, cracks/breaks, brittle/plastic deformation) within a specimen should allow for a stepwise process of retrodeformation that can be performed objectively. We recommend keeping an undeformed original mesh in the scene for reference. Furthermore, we recommend using comparative data in the form of models, photos, figures, or descriptions to inform the decision-making in anatomical reconstructions whenever possible, to minimize user bias. Description of the restoration process should accompany data in as much detail as appropriate. When retrodeformed models form the basis for analyses, sensitivity testing (of different ‘retrodeformational hypotheses’) can be useful in determining how much of an effect results from different changes and assumptions in the process of retrodeformation.

Accuracy, automation and reproducibility are relevant concerns surrounding digital fossil restoration. There are typically very few examples of a species/element to compare the accuracy of a retrodeformed model to and methods of restoration are often poorly documented [5]. Approaches using ‘armatures’ in Blender, linked with fossil fragments and ‘posed’ to correct the displacement of fragments, offer a method to document a record of manipulations [5] through saving modifications to a ‘Pose Library’ or the production of an animation. This technique is best suited to long bones (e.g. ribs, limbs). Similarly, methods to quantify deformation and characterize local and global deformation [42] improve the accuracy of retrodeformation by better understanding how specimens have been altered, as can explorations of the range and modes of taphonomic variation within species represented by frequent fossil specimens [43]. Figuring the extent of retrodeformation as tension maps of mesh distortions [5] or maps coloured by relative mesh distance between pre- and postretrodeformed conditions [44] present options for method documentation.

More automated approaches to retrodeformation have used landmarks to warp morphologies to a desired symmetry or form [32,34,42,44–47]. These approaches present an effective way to correct plastic deformation—a similar manual method in Blender might be using a Lattice modifier to correct a morphology, and Python script could be used to write more automated land-mark based retrodeformation algorithms in Blender. Nevertheless, combinations of fracturing, displacement, and both brittle and plastic deformation in specimens preclude fully automated approaches [42]. The diverse modes of damage and deformation present across fossil specimens and the importance of biomechanical data from complex multielement structures such as crania make the adaptability of Blender’s manual tools highly relevant and powerful.

### 4.2. Muscle reconstruction tool

Our Blender approach enabled the creation of similar muscles as created by the Avizo approach [28]. The muscles forces for the Avizo muscles have been published in [28]. In the Avizo approach it is easier to grow the muscles until they meet: The region grow tool built into the Avizo segmentation editor allows increasing the margin of a segmented material uniformly by one pixel. This can be done for a single slice or applied to the whole dataset. The latter effectively grows the surface by one voxel and can be applied successively as necessary. In Blender such growth would also be possible by iterative radial scaling of muscles—alternatively (or additionally), Blender muscles can be deliberately made to intersect and Boolean intersections can be used to subtract the regions of intersection. Future method development could add features to equally grow muscle bellies in the Blender method until they meet (i.e. increasing scale of muscle belly edge loops incrementally and querying intersections at each step). If other non-muscular soft-tissue structures (e.g. fat, connective tissue, neurovasculature) are present between certain muscles, these structures can also be modelled and included in the Boolean intersection calculations.

Our quantitative comparison with the frustum approach showed different muscle volumes and lengths, with the extent of this difference varying between different muscles. In addition to allowing more complex three-dimensional geometries, our Blender approach also included removing intersections between muscles and muscle and bone, which is not accounted for in the frustum approach. As expected, curving muscles such as the mPTv differ the most in volume and muscle length between the frustum and Blender three-dimensional volumetric approach. We tested two frustum calculations for the mPTv, one including the original attachments with an insertion only on the lateral mandible, and one using the attachment areas created from the bone–muscle interface after the three-dimensional muscle was created. The former method gave a frustum volume that was the most different from the three-dimensional model. For straighter, more cylindrical muscles (mAMES), the frustum approach approximates the more detailed three-dimensional Blender modelling approach. This could be due to two factors: (i) these muscles are generally more frustum-shaped and/or (ii) factors reducing muscle volumes in the three-dimensional approach (for example boolean intersections to prevent muscle–muscle and muscle–bone intersections) are countered by factors increasing the muscle volume (e.g. modelling slightly more curved muscles and somewhat increased muscle bellies relative to attachments, which would result in an increase in volume). Therefore, the research question and degree of fidelity required for the muscle will dictate which approach is most suitable. Note that our method is less objective than the frustum approach; so while it can facilitate more realistic muscles, there may be other cases in which a consistent estimate of muscle geometries between taxa is more important than more realistic muscles. Our approach enables higher fidelity (given a known three-dimensional morphology) but at the cost of time compared to the frustum approach, while still being much more time efficient than the Avizo approach. Note that in the Avizo method, the muscle length (used to derive cross-sectional area and thereby force) is also measured linearly [29], as in the frustum approach, but using manual measurements; thereby both approaches will underestimate the length of curved muscles compared to our Blender approach (in an earlier iteration of the Avizo approach, Lautenschlager [28] used the Avizo statistics module to calculate muscle cross section, but that approach assumes that all muscles are aligned with the scan axes, which is often not the case).

Holliday *et al.* [48] compared the volumes obtained from the frustum approach, the Avizo approach, and segmented muscles, and found that the Avizo approach produced similar volumes to the segmentation (more similar than the frustum approach) except for the mPTd. As noted by Holliday *et al.* [48], the mPTv and mPTd are more prone to subjectivity since they are less constrained by the surrounding bones than the other muscles.

Since both muscle length and volumes can change depending on the approach used, and both of these metrics influence muscle force calculation, we also compared the forces resulting from the different approaches (table 2). The mAMEP was the most similar between the two—like the mAMES described above, it is a roughly cylindrical muscle. However, while the mAMES volume was the most similar in the approaches, it differed more in length, highlighting the need to compare both changes in length and volume measurement between the two approaches. The mPV differed the most between the two approaches, especially when the updated attachments (both medial and lateral insertions) were used. Using both medial and lateral attachments seems more ‘realistic’ at first—however, since they are roughly parallel to each other, using them both as the insertion area in the frustum approach creates an artificially inflated muscle volume. Using only the lateral insertion reduced the difference between the frustum and Blender three-dimensional model force calculations from −74.5% to 16.2%. Therefore, if choosing to use the frustum approach for the mPTv, a lateral insertion only is a better approximation of the three-dimensional muscle volume.

Note that all of these approaches still rely on user inferences about the geometry of the muscles. Here, we attempted to reconstruct the muscles realistically, but the main goal of this study was to provide a method comparison.

#### 4.2.1. Limitations and notes on implementation

If lots of muscle shape alterations are conducted after creating the muscle mesh (e.g. sculpt tool and mesh editing), then the muscle origins and insertions and muscle curvature might change. While our tool will recalculate the volumes after such adjustments, the attachment areas and muscle length (based on the muscle curvature) are calculated during the process of muscle creation. Therefore, if the user wants to use these latter metrics in their analyses (for example by calculating muscle cross-sectional area using the curved muscle length), we encourage the users to model the muscle as accurately as possible in the first steps—if excessive muscle morphology changes are made post mesh-conversion of the muscle, the length and attachment metrics might not reflect the muscle anatomy anymore. For example, double check that the final muscle volume still aligns with origin and attachment areas if these areas are used in any analysis, and designate the desired curvature while creating the muscle. To enable mesh adjustments without losing the base geometry (curvature and attachments), the muscle meshes created with our method consist of edge loops that can easily be selected to change muscle cross sections via scaling without changing the central curvature of the muscle or the origin and insertion areas. Please see out Github README file (linked in Data accessibility) for important instructions about applying the tool.

It is important to note that by facilitating greater precision and detail in three-dimensional muscle modelling, our method has the potential to increase accuracy in muscle reconstructions, but this is only the case if the three-dimensional muscle structure is known (in the case of extant animals) or can be inferred (for extinct animals). Our goal is to provide a method that can be quickly and easily implemented, but it does not, in itself, substantiate a specific muscle shape; instead, these muscle shapes must be determined by the researchers based on their research questions and lines of evidence. Our tool also enables the option of comparing the effects of different hypothetical muscle shapes.

In this study, we assumed parallel-fibred muscles, and assumed that muscle length is equal to fibre length. Note that this is not always the case, even for parallel-fibred muscles [49,50]. Because we kept these parameters the same between muscle modelling methods, we were able to test the effects of three-dimensional muscle morphology and curved muscle lengths (versus using frusta and straight muscle lengths) on PCSA and force results between the methods. However, pennation angle and the relationship between fibre length and muscle length can affect the accuracy of the absolute muscle force outputs [19,49,50]. Therefore, the force outputs in our study are to be used to examine the relative differences between the methods, not as an absolute ground truth.

For this reason we do not include PCSA or force output as outputs of our plugin, since calculating these parameters from muscle volume depends on muscle fibre length and pennation. Researchers can then input the appropriate values for their model, for example by using data from extant animals (e.g. Porro *et al.* [51] for measurements of fibre lengths in *Alligator*). Bates & Falkingham [49] showed that in masticatory muscles, the relationship between muscle fibre length and muscle length is variable, between approximately 0.7 and 0.9 (in studies plotting fibre length against muscle belly length) and between approximately 0.57 and 0.9 (in studies plotting muscle fibre against musculotendon unit length) for a parallel fibred muscle. In reconstructions of fossil masticatory muscles, the muscle length is usually calculated from bone to bone, ignoring any tendon or aponeurosis contributions. Future studies could use our method to test modelling soft tissues such as the bodenaponeurosis in reptiles, and how this affects muscle force calculations in fossil reconstructions.

The advantage of our method is that, for whatever inputs of muscle pennation and fibre length, our method incorporates more detailed three-dimensional volumes, as well as enabling measurements of curved muscle lengths (or musculotendon unit lengths, depending on if tendons are incorporated in the model). This prevents the ‘clipping artefacts’ present in the frustum approach [48], in which the muscles are modelled without considering wrapping behaviour and bony intersections. Theoretically, using curved muscle lengths (if the three-dimensional muscle anatomy is validated) should provide a more realistic representation of muscle force (for wrapping muscles such as the mPTv), since the PCSA calculated from volume is then perpendicular to the muscle line of action (which will result in a muscle force output along the muscle line of action). Furthermore, the direction of the muscle force itself can be more accurate (again, assuming the three-dimensional reconstruction is valid), which is relevant for analyses such as FEA. Future studies can validate how modelling curved muscles affect the relationship between measured and modelled bite forces.

Notably, a recent study [52] on limb muscles demonstrated that overall muscle mass, rather than internal muscle architecture, was the best predictor for differences in whole-limb force generation. This is encouraging for fossil reconstructions, especially for studies comparing relative biomechanics (e.g. force production, stress distribution) between related species; if muscle mass can be reconstructed with a certain degree of confidence, then perhaps inter-species comparisons can be made without having to account for possible inter-species variations in fascicle length and fibre lengths (although note differences between broader groups of animals in Bishop *et al.* [52]). Further studies are needed to test if such scaling trends hold true for jaw muscles.

#### 4.2.2. Comparisons with other methods

Our muscle modelling method adds to a growing number of tools available for palaeontologists for three-dimensional muscle reconstructions [22,24,25,28]. In terms of user adjustability of muscle geometry, the method by Demuth *et al.* [25] is the most similar to ours. However, there are some notable differences. The Demuth *et al.* [25] method is based on placing vertices on the attachment areas using a ‘live surface’, whereas in our method the user simply draws around the desired attachment area and this region is automatically duplicated as the attachment area object. Furthermore, our method is based on an automatically generated curve between origin and insertion centroids which can then be adjusted to get the desired curvature, and the origin cross section is extruded along this curve automatically (again, with ability to adjust the geometry). The Demuth *et al.* [25] method is based on extruding edge loops in space manually to create the volume, and the curve length is generated after the muscle model is complete. Generally, our method is more automated in terms of the muscle modelling as well as the automatic export of outputs such as origin and attachment areas and centroids, curve length, muscle volumes to a .csv file for all muscles generated in the scene. Both models enable interactive user adjustments of the muscle geometry.

Furthermore, their method is applied in Maya (Autodesk, San Rafael, CA, USA), whereas our method is applied in Blender. We believe that the range of muscle modelling methods available in various programmes is an asset to the field, enabling researchers to choose their method and programme of preference.

#### 4.2.3. Future directions

The three-dimensional muscles can be used to directly generate more detailed muscles for finite element and multibody dynamics models. For example, the outputs of our method can be used to generate curved muscle strands which can be used as lines of action in musculoskeletal models [53,54]. This method requires muscle origin and insertion boundaries (with vertices ordered in sequence) and the muscle volume; all of these objects are generated automatically by our tool. Furthermore, our volumes could be used for fibre-embedded finite-element muscle meshes [55,56].

For studies examining extant taxa including humans, the add-on could be expanded to incorporate muscle volumes from segmented imaging data as templates. Individual-specific models could then be generated by morphing these muscle templates to the individual’s bony anatomy (for example by drawing on origin and insertion areas, or scaling the template to a scan image of the muscle cross section of the individual). Such morphing could enable subject-specific models without requiring full imaging and segmentation of each individual’s muscles; similar to statistical shape modelling (e.g. Lorenz & Krahnstöver [57], Salhi *et al.* [58], Grant *et al.* [59]).

## 5. Conclusion

In summary, here we present a guide for retrodeforming fossil specimens in Blender. We also developed an open-access, open-source Blender plug-in to enable researchers to create detailed three-dimensional muscles. These methods contribute to the ever-growing computational toolset available to palaeontologists to reconstruct form and function in extinct animals.

## Data Availability

The code for the Blender ‘Myogenerator’ add-on is available on GitHub: https://github.com/evaherbst/MyoGenerator and has been archived within the Zenodo repository: doi:10.5281/zenodo.6914448. If using this add-on, please cite this paper in any resulting publications, as well as the Zenodo DOI for the code release. A tutorial video for using the add-on can be found on Dryad at: https://doi.org/10.5061/dryad.qjq2bvqk2 [60]. Electronic supplementary material is available online at [61].
